# Editorial: Sex Determination and Developmental Mechanism of Crustacean and Shellfish

**DOI:** 10.3389/fendo.2022.940144

**Published:** 2022-06-28

**Authors:** Shubo Jin, Chao Bian, Jie Ma, Pengchao Wang, Pao Xu, Hongtuo Fu

**Affiliations:** ^1^ Key Laboratory of Freshwater Fisheries and Germplasm Resources Utilization, Ministry of Agriculture and Rural Affairs, Freshwater Fisheries Research Center, Chinese Academy of Fishery Sciences, Wuxi, China; ^2^ Shenzhen Key Lab of Marine Genomics, Guangdong Provincial Key Lab of Molecular Breeding in Marine Economic Animals, BGI Academy of Marine Sciences, BGI Marine, BGI, Shenzhen, China; ^3^ Department of Fish and Wildlife Resources, University of Idaho, Moscow, ID, United States; ^4^ Wuxi Fisheries College, Nanjing Agricultural University, Wuxi, China

**Keywords:** sex-determination, development, crustacean, shellfish, genes

## Introduction

**Figure 1:**
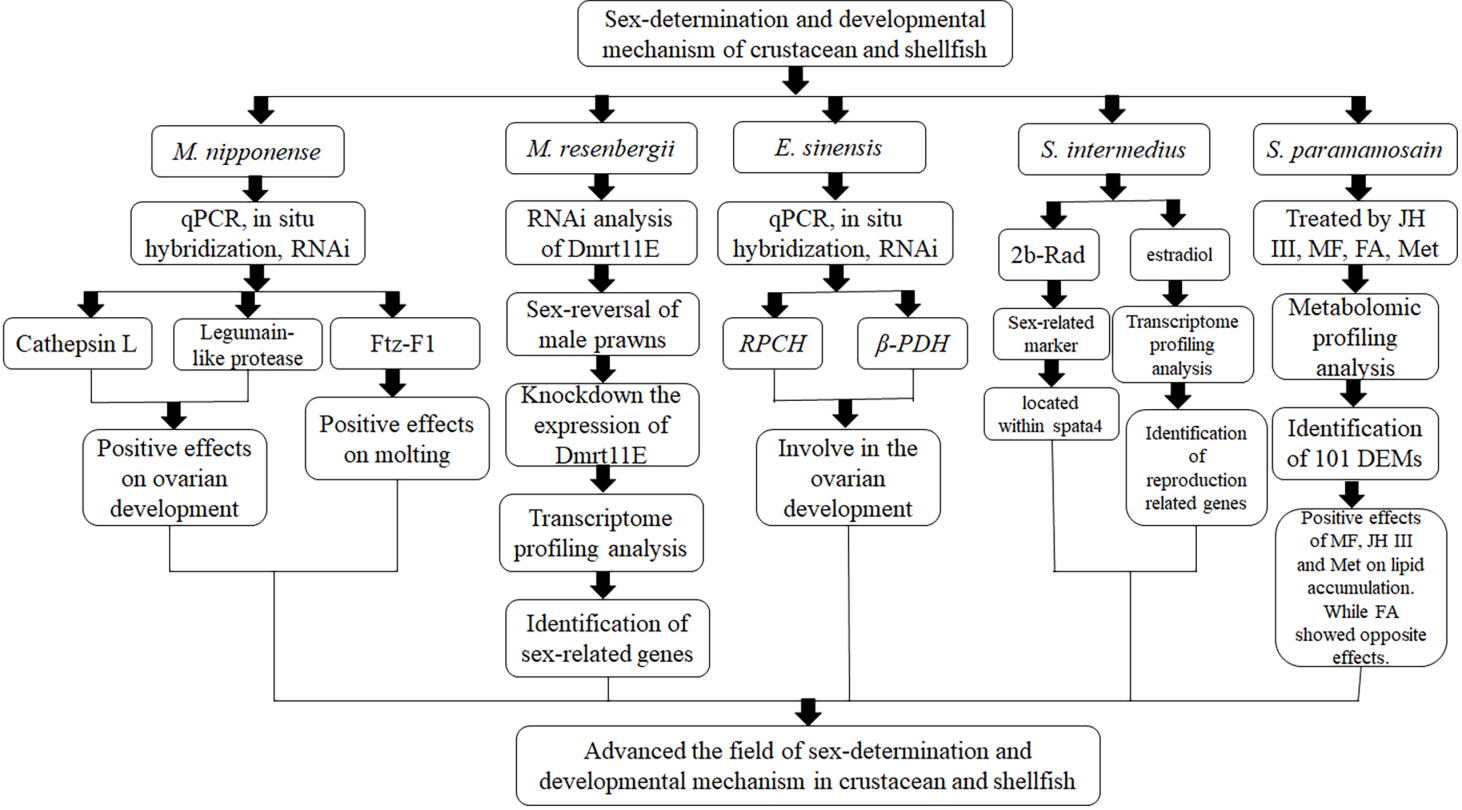
The main article contents of this Research Topic.

Culture of crustacean and shellfish species produce huge economic benefits (1). Sex-determination and reproduction are complex mechanisms in crustacean and shellfish species. Many crustaceans and shellfish show significant growth differences between male and female individuals, including *Macrobrachium nipponense* (2), *Macrobrachium resenbergii* (3), and *Eriocheir sinensis* (4). Thus, single-sex production may have dramatic economic benefits. In addition, the process of gonad maturation has a great effect on the sustainable development of aquatic animals. Slow gonad development will extend the breeding cycle, while rapid gonad development will result in the inbreeding between the new-born animals (5). Thus, the mechanisms of sex-determination and reproduction are urgently needed to be fully understood, in order to establish the technique to produce a single-sex population and regulate the gonad development in crustaceans and shellfish. The aims and objective of this Research Topic is to present an overview of the fundamental discoveries in the field of sex-determination and reproduction in crustaceans and shellfish species. The article contents of this Research Topic are shown in **Figure 1**.

## Analysis of Reproduction-Related Genes in *M. nipponense*

A total of three reproduction-related genes were analysed in *M. nipponense* by using qPCR and RNAi analysis, including Cathepsin L (*CL*), Legumain-like protease (*Lel*) and *Ftz-f1*. *CL* and *Lel* were predicted to participate in the process of ovarian development in *M. nipponense* through the transcriptome profiling analysis of five ovarian stages (6). qPCR analysis revealed that both of *Mn-Lel* and *Mn-CL* were specifically highly expressed in the hepatopancreas and ovaries of female prawns in different mature tissues. *Mn-Lel* showed significantly high expressions after metamorphosis during different development stages, while *Mn-CL* expression reached the peak before ovarian maturation in both hepatopancreas and ovary during the different ovarian developmental stages. The expressions of *Mn-Lel* were significantly decreased at day 9 and day 17 after the injection of *Mn-Lel* dsRNA in both hepatopancreas and ovary, and changes in the gonad somatic index (GSI) confirmed the inhibitory effects of *Mn-Lel* dsRNA on ovary maturation (Jiang et al.). The expressions of *Mn-CL* were significantly decreased at day 9 and day 17 after the injection of *Mn-CL* dsRNA in ovary, and the expression of *Mn-vitellogenin* were also decreased with the decrease of *Mn-CL*, indicating cathepsin L has positively regulatory roles together with that of vitellogenin in *M. nipponense*. Changes in the GSI also confirmed the inhibitory effects of *Mn-CL* dsRNA on ovary maturation (Jiang et al.). These results indicated that both cathepsin L and Legumain-like protease have positively regulatory roles on ovary maturation in *M. nipponense*.

*Mn-Ftz-f1* expression showed the highest expression level in ovary, based on the PCR analysis in different mature tissues, and *Mn-Ftz-f1* expression reached the peak at the larval developmental stage 5 (L5) during the different developmental stages, followed by the post-larval developmental stage 5 (PL5). RNAi analysis revealed that the injection of *Mn-Ftz-f1* dsRNA significantly decreases the expression of *Mn-Ftz-f1* in *M. nipponense*, and the expressions of the vitellogenin, Spook, and Phantom genes were decreased with the decrease of *Mn-Ftz-f1* in *M. nipponense*, indicating that *Ftz-f1* positively regulated the expressions of these genes in *M. nipponense*. In addition, the molting frequency and ovulation number of *M. nipponense* decreased significantly after the injection of *Mn-Ftz-f1* dsRNA, demonstrating that *Mn-Ftz-f1* has positive effects on the process of molting and ovulation (Yuan et al.).

## Identification of Sex-Determination-Related Genes From *M. rosenbergii*

*In vivo* knockdown of the expressions of Dmrt11E by RNAi at the post-larva stage in male *M. rosenbergii* results in a complete and functional sex reversal and production of an all-male monosex population. The sex-determination-related genes were further investigated from *M. rosenbergii* through performing the transcriptome profiling analysis after the injection of Dmrt11E dsRNA. Transformer, fruitless, feminization, insulin-like androgenic gland gene, and Dmrt gene family were primarily identified to be regulated by the changes of Dmrt11E, which were predicted to be involved in the mechanism of sex-determination in *M. rosenbergii* (Xu et al.).

## Identification of the Functions of Reproduction-Related Genes in *E. sinensis*

The potential functions of the red pigment concentrating hormone (RPCH) and the pigment dispersing hormone (PDH) on the ovarian development were investigated in *E. sinensis*. qPCR analysis identified that *Es-RPCH* and *Es-β-PDH* transcripts were distributed in the brain and eyestalks. The *in vivo* injection of *Es-RPCH* and *Es-β-PDH* peptides into the female *E. sinensis* results in the significant decrease of GSI and mean oocyte diameter, as well as the expression of vitellogenin, cdc2 kinase, cyclin B, 5-HT-R and retinoid-X receptor mRNA, indicating that *Es-RPCH* and *Es-β-PDH* have the inductive effects on the oocyte meiotic maturation in *E. sinensis* (Wei et al.).

## Identification of the Effect of Estradiol on *Strongylocentrotus intermedius*

A sex-associated single nucleotide polymorphism (SNP) was identified in *S. intermedius*, which was located within spata4. Knockdown the expressions of spata4 by RNAi results in the significant changes of the other well studied testis differentiation-related genes and germ cell marker genes, including *Dmrt1*, *SoxE*, *Foxl2*, *Hsd17b8*, *Boule* and *Nanos1*. In addition, the differentially expressed genes affected by estradiol were identified through performing the transcriptome profiling analysis after a 58-day feeding experiment, which may be involved in the process of testis differentiation, ovary differentiation, and estrogen metabolism in *S. intermedius* (Han et al.).

## Identification of Metabolites Affecting the Ovarian Development of *Scylla paramamosain*

A total of 101 differentially expressed metabolites were identified after the treatment with juvenile hormone III (JH III), methyl farnesoate (MF), farnesoic acid (FA) and methoprene (Met), enriched in 47 metabolic pathways, providing a comprehensive insight to understand the process of ovarian development in *S. paramamosain*. MF, JH III and Met played an extensive role in lipid accumulation, while FA has opposite effects (Fu et al.).

## Author Contributions

SJ wrote the editorial. CB, JM, and PW summarize the findings of the articles in this Research Topic. PX and HF supervised and revised the editorial. All authors contributed to the article and approved the submitted version.

## Funding

This research was supported by grants from the National Key R&D Program of China (2018YFD0900201); Central Public-interest Scientific Institution Basal Research Fund CAFS (2021JBFM02; 2020TD36); Jiangsu Agricultural Industry Technology System; the China Agriculture Research System-48 (CARS-48); the New cultivar breeding Major Project of Jiangsu province (PZCZ201745).

## Conflict of Interest

The authors declare that the research was conducted in the absence of any commercial or financial relationships that could be construed as a potential conflict of interest.

## Publisher’s Note

All claims expressed in this article are solely those of the authors and do not necessarily represent those of their affiliated organizations, or those of the publisher, the editors and the reviewers. Any product that may be evaluated in this article, or claim that may be made by its manufacturer, is not guaranteed or endorsed by the publisher.
